# Supplementary material for: Removal of detritivore sea cucumbers from reefs increases coral disease
Source: Nat Commun. 2024 Feb 26;15:1338. doi: 10.1038/s41467-024-45730-0 (PMC10897328; doi:10.1038/s41467-024-45730-0)
Supplement: Supplementary file 1 — Supplementary Information [file 41467_2024_45730_MOESM1_ESM.pdf]

Removal of detritivore sea cucumbers from reefs increases coral disease

Cody S. Clements[1], Zoe A. Pratte[2], Frank J. Stewart[2], Mark E. Hay[1*]

[1] School of Biological Sciences and Center for Microbial Dynamics and Infection; Georgia Institute of Technology, Atlanta, GA 30332–0230, USA

[2] Department of Microbiology and Cell Biology; Montana State University, Bozeman, MT 59717-3520, USA

*Corresponding author. Email: mark.hay@biology.gatech.edu

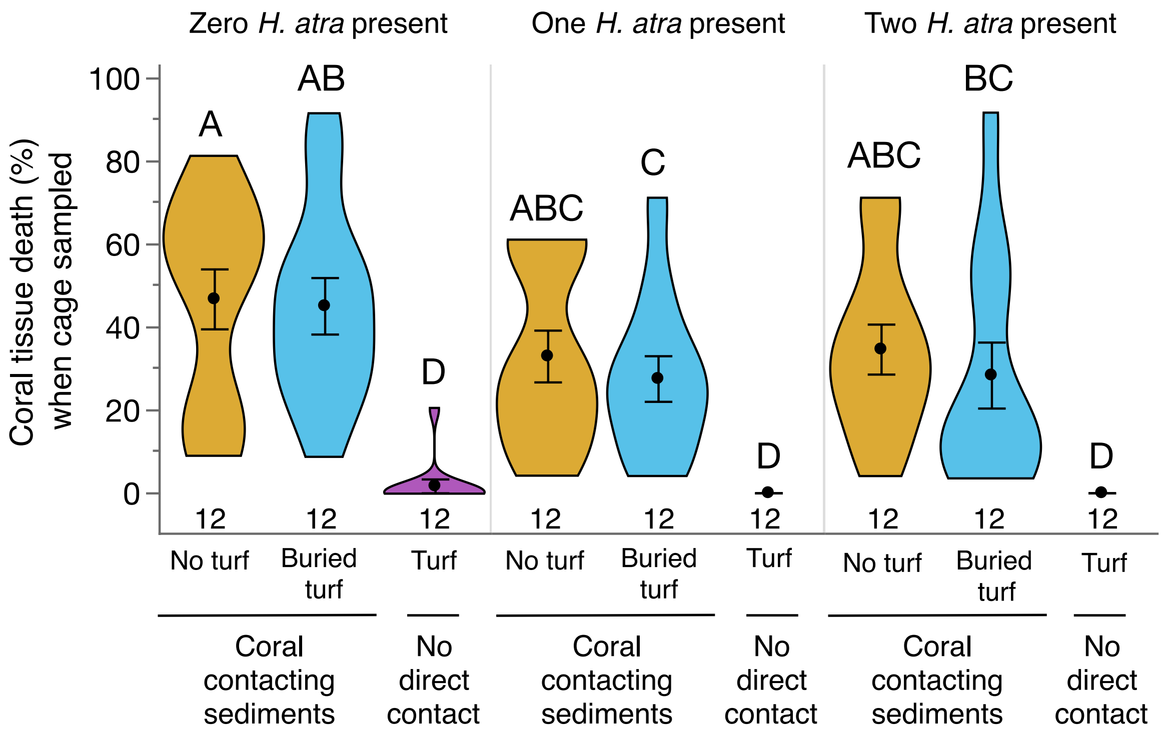

Supplementary Figure 1: *A. pulchra* percent tissue death (mean ± SE) as a function of *H. atra* density (zero, one, or two individuals; $p = 0.014$) and coral outplant type (no turf, buried turf, or turf; $p = 0.001$). Outplants with no turf and buried turf were planted so that living coral tissue directly contacted the sediment, while turf samples reflect living coral tissue that was separated from the sediment by farmerfish turf algae on the base of the outplant. Total numbers of coral outplants assessed per treatment are indicated below each violin plot. *P* values derived from a permutation-based linear mixed-effects (LME) model. Letters indicate significant groupings via a post hoc permutation test for multiple comparisons.

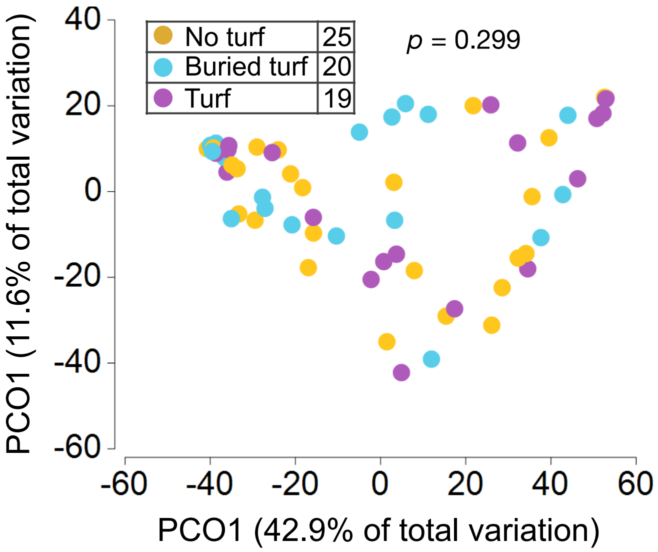

Supplementary Figure 2: PCoA (Bray-Curtis dissimilarity index) and PERMANOVA analysis of microbiome composition (beta diversity) of distal sampling locations based on coral outplant type. Outplants with no turf and buried turf were planted in such a way that living coral tissue directly contacted the sediment, while turf samples derived from living coral tissue that was separated from the sediment by basal turf algae. Total numbers of samples assessed per treatment are indicated within the legend.
