# Supplementary material for: Removal of detritivore sea cucumbers from reefs increases coral disease
Source: Nat Commun. 2024 Feb 26;15:1338. doi: 10.1038/s41467-024-45730-0 (PMC10897328; doi:10.1038/s41467-024-45730-0)
Supplement: Supplementary file 2 — Reporting Summary [file 41467_2024_45730_MOESM2_ESM.pdf]

Corresponding author(s): Mark Hay
Last updated by author(s): Feb 1, 2024

Reporting Summary

Nature Portfolio wishes to improve the reproducibility of the work that we publish. This form provides structure for consistency and transparency in reporting. For further information on Nature Portfolio policies, see our Editorial Policies and the Editorial Policy Checklist.

Statistics

For all statistical analyses, confirm that the following items are present in the figure legend, table legend, main text, or Methods section.

n/a	Confirmed	
☐	☒	The exact sample size (*n*) for each experimental group/condition, given as a discrete number and unit of measurement
☐	☒	A statement on whether measurements were taken from distinct samples or whether the same sample was measured repeatedly
☐	☒	The statistical test(s) used AND whether they are one- or two-sided<br>*Only common tests should be described solely by name; describe more complex techniques in the Methods section.*
☐	☒	A description of all covariates tested
☐	☒	A description of any assumptions or corrections, such as tests of normality and adjustment for multiple comparisons
☐	☒	A full description of the statistical parameters including central tendency (e.g. means) or other basic estimates (e.g. regression coefficient) AND variation (e.g. standard deviation) or associated estimates of uncertainty (e.g. confidence intervals)
☐	☒	For null hypothesis testing, the test statistic (e.g. *F*, *t*, *r*) with confidence intervals, effect sizes, degrees of freedom and *P* value noted<br>*Give P values as exact values whenever suitable.*
☒	☐	For Bayesian analysis, information on the choice of priors and Markov chain Monte Carlo settings
☒	☐	For hierarchical and complex designs, identification of the appropriate level for tests and full reporting of outcomes
☒	☐	Estimates of effect sizes (e.g. Cohen's *d*, Pearson's *r*), indicating how they were calculated

Our web collection on statistics for biologists contains articles on many of the points above.

Software and code

Policy information about availability of computer code

Data collection	No software was used for data collection.
Data analysis	Analyses for data used in this paper are outlined below.<br><br>For data collected from the experiment conducted in natural sand patches in Moorea (see “Study areas and natural sand patch experiments” in the Methods section of the manuscript):<br><br>We used permutation-based, linear mixed-effects (LME) model in the R package predictmeans to compare differences in percent tissue mortality between corals in plots where sea cucumbers were removed versus not removed. Patch type (removed vs. not removed) was treated as a fixed factor, with replicate patches treated as a random effect nested within patch type. To compare overall coral survivorship at 45 days, survival curves and analyses were generated using random-effects Weibull regression in Stata (version 17), with patch type as a fixed effect and replicate patches treated as a random effect nested within patch type.<br><br>For data collected from the sea cucumber enclosure experiment in Moorea (see “Sea cucumber enclosure experiments” in the Methods section of the manuscript):<br><br>We used a permutation-based, linear mixed-effects (LME) model in the R package predictmeans. Sea cucumber status and coral outplant type were treated as fixed effects and cage as a random effect. Subsequent comparisons were conducted using a post hoc permutation test for multiple comparisons using predictmeans. We used Fisher’s exact tests to assess differences in the frequency of mortality-initiated sampling among sea cucumber treatments (i.e., zero, one, or two H. atra) and among outplant types (i.e., no turf, turf, or embedded turf).

For data collected from the sea cucumber enclosure experiment in Palmyra Atoll (see "Sea cucumber enclosure experiments" in the Methods section of the manuscript):

We used a permutation-based, linear mixed-effects (LME) model in the R package predictmeans to compare differences in percent coral tissue mortality as a function of sea cucumber presence vs. absence, as well as coral direct contact vs. no contact with sediment. Sea cucumber status and coral-sediment contact status were treated as fixed effects and cage treated as a random effect. Subsequent comparisons were conducted using a post hoc permutation test for multiple comparisons using predictmeans.

For analyses of microbiome data from the sea cucumber enclosure experiment in Moorea (see "Microbiome data analyses" in the Methods section of the manuscript):

Bray-Curtis dissimilarity values were calculated using the distance function in PRIMER-e. Principal coordinate analysis (PCO) and corresponding tests for differences in microbiome composition (permutational multivariate analysis of variance, PERMANOVA) were implemented in PRIMER-e via tests with one (Q i-ii) or two (Q iii-iv) factors. If significant, subsequent comparisons were conducted using the "Pair-wise test" option within the PERMANOVA function of PRIMER-e. To test for differences in microbiome variability (dispersion, measured as deviation from the centroid), we used the PERMDISP function in PRIMER-e for all relevant analyses, including subsequent pairwise comparisons when significant. Alpha diversity (ESV richness, Shannon diversity) of relevant datasets was calculated using the core-metrics-phylogenetic function in QIIME2. Relevant comparisons were conducted using permutational ANOVA in the R package predictmeans. To identify differential ESV abundances, non-rarefied tables were imported into R through the packages qiime2R and phyloseq. In cases where pairwise comparisons were statistically indistinguishable in broader-scale microbiome metrics (e.g., microbiome composition), these non-significant sample groupings were collapsed into a single grouping, and ESV abundances were compared to those of groupings that differed significantly in broader-scale microbiome metrics. DESeq2 was then used to detect differential ESV abundance among relevant sample groupings, with a minimum adjusted p-value ≤ 0.05.

For manuscripts utilizing custom algorithms or software that are central to the research but not yet described in published literature, software must be made available to editors and reviewers. We strongly encourage code deposition in a community repository (e.g. GitHub). See the Nature Portfolio guidelines for submitting code & software for further information.

Data

Policy information about availability of data

All manuscripts must include a data availability statement. This statement should provide the following information, where applicable:

- Accession codes, unique identifiers, or web links for publicly available datasets
- A description of any restrictions on data availability
- For clinical datasets or third party data, please ensure that the statement adheres to our policy

The coral tissue mortality data generated in this study have been deposited in the BCO-DMO data system (https://www.bco-dmo.org/project/837802). Raw sequence data are available at NCBI's SRA Database under BioProject PRJNA1013970 (https://www.ncbi.nlm.nih.gov/bioproject/1013970). All data needed to evaluate the conclusions in the paper are present in the paper and/or the Supplementary Information. Source data are provided with this paper

Research involving human participants, their data, or biological material

Policy information about studies with human participants or human data. See also policy information about sex, gender (identity/presentation), and sexual orientation and race, ethnicity and racism.

Reporting on sex and gender	N/A
Reporting on race, ethnicity, or other socially relevant groupings	N/A
Population characteristics	N/A
Recruitment	N/A
Ethics oversight	N/A

Note that full information on the approval of the study protocol must also be provided in the manuscript.

Field-specific reporting

Please select the one below that is the best fit for your research. If you are not sure, read the appropriate sections before making your selection.

☐ Life sciences ☐ Behavioural & social sciences ☒ Ecological, evolutionary & environmental sciences

For a reference copy of the document with all sections, see nature.com/documents/nr-reporting-summary-flat.pdf

Ecological, evolutionary & environmental sciences study design

All studies must disclose on these points even when the disclosure is negative.

Study description

Each experiment included in this paper is described below.

For the experiment conducted in natural sand patches in Moorea (see "Study areas and natural sand patch experiments" in the Methods section of the manuscript):

We assessed the effects of the sea cucumber Holothuria atra on the coral Acropora pulchra within a shallow fringing reef on the north coast of Moʻorea, French Polynesia. We chose 20 of these patches (study area = ~8,900 m2, patch areas ranging from 6.27-12.15 m2), randomly assigned each to a removal or control plot, and either consistently removed (every ~1-2 days) or did not remove sea cucumbers to test how sea cucumber removal impacted A. pulchra tissue mortality and survivorship.
At the initiation of the experiment, sea cucumbers were removed daily (removals) or left in place (controls) for seven days to condition sand patches for subsequent coral planting, after which five A. pulchra outplants approximately 8-10 cm in length were embedded in the sediment of each patch, with % coral tissue mortality and outplant survival monitored at approximately 2-day intervals for 45 days (50 corals treatment-1, 100 corals total). Corals were embedded within their sand patches so that living basal coral tissue was in direct contact with the sediment as would occur following natural fragmentation. Every other day for 45 days, we counted sea cucumbers, maintained removal treatments, cleaned cages, and quantified A. pulchra tissue mortality in each patch. We used permutation-based, linear mixed-effects (LME) model in the R package predictmeans to compare differences in percent tissue mortality between corals in plots where sea cucumbers were removed versus not removed. Patch type (removed vs. not removed) was treated as a fixed factor, with replicate patches treated as a random effect nested within patch type. To compare overall coral survivorship at 45 days, survival curves and analyses were generated using random-effects Weibull regression in Stata (version 17), with patch type as a fixed effect and replicate patches treated as a random effect nested within patch type.

For data collected from the sea cucumber enclosure experiment in Moorea (see "Sea cucumber enclosure experiments" in the Methods section of the manuscript):

In Moʻorea, to assess the impact of sea cucumber removal on sediment- and coral-associated microbiomes, as well as how farmerfish turf on the base of corals might affect disease prevalence, we erected thirty-six 50 cm x 50 cm x 12 cm tall cages using 1 cm2 grid metal screening to contain or exclude sea cucumbers and prevent access by coral consumers. Cages were situated in an ~85 m2 sand patch within the fringing reef area utilized in our initial experiment described above and were separated from adjacent cages by ≥60 cm, creating a 6 x 6 grid of enclosures. Each cage was stocked with either zero, one, or two H. atra (12 cages treatment-1). Density treatments were assigned at random. Three A. pulchra outplants were embedded into the sediment of each cage (108 outplants total) to test the potential effects of (i) sea cucumber density, and (ii) protective effects of farmerfish-cultivated turf algae on coral health and microbiomes. These three outplants were embedded into the sediment as follows: (i) coral lacking turf planted in direct contact with benthic sediment (hereafter "no turf"), (ii) coral separated from direct contact with sediment by turf algae growing at its base (hereafter "turf"), or (iii) coral with turf on its base, but embedded more deeply into the sediment so that the living coral tissue was in direct contact with the sediment (hereafter "embedded turf"). Because each cage contained a block of all coral transplant types, this allowed us to minimize the variance due to location and focus on the response of coral outplant type. Percent coral tissue mortality among outplants was visually estimated daily for 36 days. The microbiomes of all corals and the sediment within a cage were sampled when one or more outplants within that cage exhibited ≥50% tissue mortality or when the experiment was terminated on day 36. We used Fisher's exact tests to assess differences in the frequency of mortality-initiated sampling among sea cucumber treatments (i.e., zero, one, or two H. atra) and among outplant types (i.e., no turf, turf, or embedded turf). In analyses of coral tissue mortality, we used a permutation-based, linear mixed-effects (LME) model in the R package predictmeans. Sea cucumber status and coral outplant type were treated as fixed effects and cage as a random effect. Subsequent comparisons were conducted using a post hoc permutation test for multiple comparisons using predictmeans.

For data collected from the sea cucumber enclosure experiment in Palmyra Atoll (see "Sea cucumber enclosure experiments" in the Methods section of the manuscript):

Within an area of about 160 m2, we erected thirty 50 x 50 x 12 cm cages constructed of plastic 1 cm2 mesh, planted two A. nasuta either in contact with or elevated 2-3 cm above benthic sediment into each cage (planted as described above), and randomly assigned cages to contain either zero or two S. chloronotus sea cucumbers (n = 15 for each treatment combination). We used a permutation-based, linear mixed-effects (LME) model in the R package predictmeans to compare differences in percent coral tissue mortality as a function of sea cucumber presence vs. absence, as well as coral direct contact vs. no contact with sediment. Sea cucumber status and coral-sediment contact status were treated as fixed effects and cage treated as a random effect. Subsequent comparisons were conducted using a post hoc permutation test for multiple comparisons using predictmeans.

Research sample

Sample choice: We used Acropora spp. corals (A. pulchra and A. nasuta) because these are abundant at the sites studied, the most speciose genus in the Pacific, and are responsible for generating much of the topographic complexity upon which many reef species depend. We used the two species of sea cucumbers (Holothria atra and Stichopus chloronotus) because these were the most abundant ones at each site studies (really the only ones not harvested to near extinction in the past).

Organisms: Two species of Acroporid corals (A. pulchra and A. nasuta) and two locally abundant sea cucumbers (H. atra and S. chloronotus).

Age: These are field collected individuals. The corals are clonal and ages cannot be determined by size. The sea cucumbers can under go binary fission at larger sizes, so they also cannot be aged - even by size.

Manipulation: These are field collected animals and were not manipulated artificially. Populations represented: normal wild species

	in the field.
Sampling strategy	Sample sizes were chosen based on 40 years of previous experience with field experimentation. If one has previous data to estimate variance in responses, one can "hopefully" calculate desired power and needed sample size but 1) no such previous data exist for the experiments we conducted and 2) recent statistical authorities have questioned the validity of such power analyses anyway.
Data collection	Data were recorded visually in the field as corals infected or not and % tissue mortality binned in categories from 0, 10, etc.% up to 100%. Data were recorded on waterproof paper with a pencil and then entered into relevant spreadsheets on a computer. Data assessed by Cody Clements.
Timing and spatial scale	Data represented in Figure 1c-d were collected from an experiment that was conducted 6/26/21–8/10/21 in Moorea, French Polynesia. Coral tissue mortality was assessed visually every day. The experiment ended on 8/10/21 due to time constraints of field work. Data represented in Figures 2–3 were collected from an experiment that was conducted 4/20/20–5/26/20 in Moorea, French Polynesia. Sediment in each cage was sampled on 4/20/23. Coral tissue mortality was assessed visually every two days until dead tissue on any coral in that cage reached 50% mortality. All corals in that cage were then sampled for later analyses of coral microbiomes. Data represented in Figure 1e were collected from an experiment that was conducted 11/6/22 – 11/20/22 in Palmyra Atoll. Tissue mortality was assessed visually every day. Corals and sediment in each cage were sampled 11/20/22 for subsequent microbial analyses.<br><br>Timing in Moorea was determined by availability of lodging and lab space and boat availability during times the PI was not required to be on campus teaching. Timing in Palmyra was determined by The Nature Conservancy and availability of seats on the small chartered jet that is the only way to travel to and from the island. Spatial scale varied among experiments. The experiment removing sea cucumbers from specific sand patches covered an area of approximately 4 hectares. The experiments involving inclusion and exclusion cages in Moorea and Palmyra were conducted in areas of about 30-40 square meters.
Data exclusions	No data were excluded.
Reproducibility	These were controlled, replicated, and interspersed experiments. They were conducted in two very different sites about 2000 km apart and gave similar results.
Randomization	For data represented in Figure 1c-d, 10 coral branches were fragmented from 20 colonies within the surrounding lagoonal area. Five branches from each colony were randomly outplanted into a patch where sea cucumbers were removed and the other five were outplanted to a patch where sea cucumbers were not removed. For data represented in Figures 2–3, nine coral branches were collected from 12 colonies within the surrounding lagoonal area. Three branches from each colony were randomly outplanted within cages that had zero, one, or two Holothuria atra. For data represented in Figure 1e, four coral branches were collected from 15 colonies. Two branches from each colony were randomly outplanted within cages that had zero or two Stichopus chloronotus.
Blinding	Blinding was not possible due to field conditions and personnel. For example, the researchers set-up, monitored, and sampled the experiment and thus were aware of sand patches where sea cucumbers were removed vs. not removed.

Did the study involve field work? ☒ Yes ☐ No

Field work, collection and transport

Field conditions	Normal field conditions for the site. Weather was variable, waves modest due to being in the lagoon, etc.
Location	In shallow lagoons on the north coast of Mo’orea, French Polynesia (17.4894° S, 149.8825° W) and Palmyra Atoll (5°52'42.6"N 162° 04'09.8"W).
Access & import/export	The French Polynesian Government (Délégation à la Recherche) and the Haut-commissariat de la République en Polynésie Française (DTRT) issued permits (Protocole d'Accueil 2017-2021) for us to work in French Polynesia and the US Fish and Wildlife Service and The Nature Conservancy for permits and support our work in Palmyra Atoll.
Disturbance	Cages were erected in the field and removed after the experiments. Experiments were conducted in sandy environments and thus there were no lasting effects. All corals and sea cucumbers used in field experiments were released (sea cucumbers) or replanted (corals) back into the field near their sites of collection at the end of each experiment.

Reporting for specific materials, systems and methods

We require information from authors about some types of materials, experimental systems and methods used in many studies. Here, indicate whether each material, system or method listed is relevant to your study. If you are not sure if a list item applies to your research, read the appropriate section before selecting a response.

Materials & experimental systems

n/a	Involved in the study
☒	☐ Antibodies
☒	☐ Eukaryotic cell lines
☒	☐ Palaeontology and archaeology
☐	☒ Animals and other organisms
☒	☐ Clinical data
☒	☐ Dual use research of concern
☒	☐ Plants

Methods

n/a	Involved in the study
☒	☐ ChIP-seq
☒	☐ Flow cytometry
☒	☐ MRI-based neuroimaging

Animals and other research organisms

Policy information about studies involving animals; ARRIVE guidelines recommended for reporting animal research, and Sex and Gender in Research

Laboratory animals

N/A

Wild animals

Densities of sea cucumbers (Holothria atra and Stichopus chloronotus) in both sites were manipulated by hand (put into or excluded from cages) or left in or removed from sand patches. At the end of the experiment, sea cucumbers were released from cages or allowed to return to removal patches via natural foraging movements. No animals were killed.

Corals (Acropora pulchra and Acropora nasuta) were initially fragmented from colonies at our study areas using a hammer, chisel, and/or aviation snips. Corals were individually embedded (by hand) within the cutoff necks of inverted plastic bottles using Z-Spar Splash Zone epoxy and screwed into upturned bottle caps attached to ~7 x 7 cm pieces of metal gridded mesh that could be slid into the sediment to hold each coral upright. At the end of the experiments, all living corals were outplanted back to the reef where they were collected.

In the case of sea cucumbers and corals, these are wild caught organisms, and it is not possible to determine their age. Corals are clonal and can expand, fracture and reestablish via vegetative spread, and shrink due to past damage. Thus large individuals can be 50-100+ years old, but so can small individuals. For some similar reasons, sea cucumbers collected in the field also cannot be aged. Larger individuals of these species can split to form 2 smaller individuals so even based on size one cannot reasonable estimate. We listed the sizes used in the text of the MS.

Reporting on sex

N/A

Field-collected samples

Sediment and coral (0.5g clipping) samples for microbiome analyses. Sediment samples were immediately placed on ice when collected in the field and stored in a -80°C freezer upon return to shore. Samples remained frozen until processed (DNA extraction and sequencing). Coral samples were placed in 2 ml cryovials with RNA/DNA stabilizing buffer (25mM sodium citrate, 10mM EDTA and 70g ammonium sulfate per 100ml solution, pH 5.2) when collected in the field, immediately placed on ice, and stored in a -80°C freezer upon return to shore. Samples remained frozen until processed (DNA extraction and sequencing).

Ethics oversight

None were required since our experiments did not use vertebrates (sea cucumbers and corals are invertebrates).

Note that full information on the approval of the study protocol must also be provided in the manuscript.

Plants

Seed stocks

N/A

Novel plant genotypes

N/A

Authentication

N/A
