# Supplementary material for: Removal of detritivore sea cucumbers from reefs increases coral disease
Source: Nat Commun. 2024 Feb 26;15:1338. doi: 10.1038/s41467-024-45730-0 (PMC10897328; doi:10.1038/s41467-024-45730-0)
Supplement: Supplementary file 4 — Description of Additional Supplementary Files [file 41467_2024_45730_MOESM4_ESM.pdf]

Description of Additional Supplementary Files

File Name: Supplementary Data 1
Description: Basal coral sample ESVs that differ significantly in abundance between coral outplants contacting sediment (no turf and buried turf treatments) or separated from sediment by farmerfish turf algae.

File Name: Supplementary Data 2
Description: Sediment sample ESVs that differ significantly between the treatment with zero versus 2 sea cucumbers.

File Name: Supplementary Data 3
Description: Basal coral samples evaluated at the resolution of 90% similarity (roughly genus level) that differ significantly in abundance between coral outplants contacting sediment (no turf and buried turf treatments) or separated from sediment by farmerfish turf algae.

File Name: Supplementary Data 4
Description: Sediment samples evaluated at the resolution of 90% similarity (roughly genus level) that differ significantly between the treatment with zero versus 2 sea cucumbers.

File Name: Supplementary Movie 1
Description: Aerial videos of the back reef lagoon of Moʻorea showing abundant sea cucumbers (*Holothuria atra*, red circles denote example individuals).

File Name: Supplementary Movie 2
Description: *Acropora pulchra* outplants experiencing the typical pattern of tissue mortality from the base up when in direct contact with sediments (magnified in red circles). Video footage comes from enclosures where sea cucumbers were absent.
